# Long-term masticatory performance and ability following closed treatment for unilateral mandibular condylar neck or base fractures: a cross-sectional study

**DOI:** 10.1007/s10006-021-01027-w

**Published:** 2022-01-23

**Authors:** Florine M. Weinberg, Antoine J. W. P. Rosenberg, Barbara S. Muller, Caroline M. Speksnijder

**Affiliations:** Department of Oral and Maxillofacial Surgery and Special Dental Care, University Medical Center Utrecht, Utrecht University, G05.122, P.O. Box 85.500, 3508 GA Utrecht, The Netherlands

**Keywords:** Mandibular condyle, Masticatory performance, Masticatory ability, Mixing ability test

## Abstract

**Purpose:**

The aim of this study was to find explanatory variables for objective and patient-reported long-term masticatory functioning in patients treated with maxillomandibular fixation for unilateral condylar neck or base fractures. These outcomes were compared to healthy control subjects.

**Methods:**

Patients treated between 1996 and 2013 were enrolled in the study. Objective measurements included the mixing ability test (MAT) for masticatory performance, and range of motion of the mandible. Patient-reported measurements included the mandibular function impairment questionnaire (MFIQ) for masticatory ability, and the visual analogue scale for pain. Healthy subjects were recruited between October 2018 and January 2019, and performed the MAT and MFIQ.

**Results:**

Twenty-one patients and 30 healthy subjects were included. The average follow-up period was 11.67 years. In adjusted regression analysis, the amount of occlusal units (OU) was associated with the MAT (*P* = 0.020; R^2^ = 0.253) and MFIQ (*P* = 0.001, R^2^ = 0.454). The MAT outcome was similar in both groups when correcting for OU (*P* = 0.001; R^2^ = 0.201). The MFIQ was inferior in the patient group (*P* = 0.001).

**Conclusion:**

Long-term masticatory performance was similar in patients with a history of condylar neck or base fracture and healthy subjects; however, masticatory ability was inferior in patients compared to healthy subjects.

## Introduction

The condyle is one of the most common sites of mandibular fracture, accounting for between 16 and 43% of fractures [1]. Traditionally, the management of mandibular condylar fractures involves closed treatment, where occlusion is corrected by maxillomandibular fixation (MMF) with either wires or elastics. Although this procedure is often preferred over open reduction with internal fixation (ORIF), the management of condylar fractures remains a subject of ongoing debate [2, 3]. The primary goal in condylar fracture treatment is the restoration of occlusion. The secondary goals are to optimise patient outcome, oral functioning and masticatory problems, in particular, which are frequently observed following maxillofacial injury [4, 5]. Rehabilitation of masticatory deficits after condylar trauma can be measured through masticatory performance and masticatory ability [6–8]. One of the main factors in determining masticatory performance is the number of occluding units (OU). This in combination with bite force could explain 70% of the variance in masticatory performance.[9] Masticatory performance can be objectively measured with the mixing ability test (MAT), which assesses the comminution of a bolus over a standard number of chewing cycles [6–8]. Masticatory ability can also be subjectively measured through patients’ own perception of mastication, assessed using questionnaires such as the mandibular function impairment questionnaire (MFIQ) [6–8].

To the best of our knowledge, no studies in the literature have focussed on masticatory performance, assessed by MAT, and masticatory ability, evaluated by MFIQ, in patients following condylar fracture with a follow-up of at least 5 years. These long-term results are clinically valuable, as they can guide treatment decision-making and determine appropriate follow-up periods in this population [4, 10]. The first aim was to find explanatory demographic and clinical variables for masticatory performance and ability in patients who received closed treatment for unilateral condylar neck or base fractures at least 5 years ago. The second aim was to compare masticatory performance and masticatory ability between patients with a history of condylar fractures and healthy subjects.

## Materials and methods

### Patients and healthy subjects

Patients with unilateral fractures of the condylar neck or base who received closed treatment with MMF at the Department of Oral and Maxillofacial Surgery of the University Medical Center Utrecht (UMCU) between January 1996 and December 2013 were enrolled in the study. The condylar fractures were classified in neck or base fractures according to the AOCMF classification system published in 2014 [11]. The exclusion criteria were as follows: (1) younger than 18 years of age at the time of trauma, (2) unable to understand and/or read Dutch, (3) presence of a bilateral condylar fracture or additional fracture of the midface, (4) reported intellectual disability or a history of psychiatric disorder(s), and (5) presence of condylar head fractures according to the AOCMF classification system for condylar process fractures [11]. This study followed the Declaration of Helsinki on medical protocol and ethics, and the Ethics Committee of the UMCU approved the study protocol (NL600.70.041.17). All patients who met the inclusion criteria were invited by letter to participate in a one-time visit to the outpatient clinic. If patients did not respond within 3 months, a follow-up letter was sent. All participants signed an informed consent form.

Data collection and reporting were based on the STROBE Statement checklist for cross-sectional studies [12]. The following demographic data were collected: gender, current age, age at the time of trauma, cause of trauma, diagnosis, other mandibular fractures, type of MMF received (guiding elastics or wires), total duration of treatment, and complications classified according to the Clavien-Dindo classification (CDC) [13]. The following data were collected: active range of motion (AROM) of the mandible, including active maximum mouth opening (MMO), laterotrusion and protrusion; symptoms (clicking, pain, crepitation; yes or no) of the temporomandibular joint (TMJ); occlusion (stable occlusion, patient-reported malocclusion, objectively measured malocclusion); and OU. The number of OU was assessed as the functional units of the patients natural dentition in the premolar and molar region (range 0–12), where an occluding pair of premolars counts for one, and an occluding pair of molars counts for two. Additionally, pain at rest was assessed through a visual analogue scale (VAS). The mixing ability test (MAT) was performed to measure masticatory performance. Masticatory ability was evaluated through the mandibular function impairment questionnaire (MFIQ).

Healthy subjects were recruited between October 2018 and January 2019. The exclusion criteria were as follows: (1) younger than 18 years of age, (2) unable to apprehend and/or read Dutch, (3) functional disorders of the head and neck region, (4) reported intellectual disabilities or a history of psychiatric disorder(s), and (5) history of facial trauma. The Ethics Committee of the UMCU approved the study protocol (18–701/C). All healthy subjects were randomly asked to participate when they visited our outpatient clinic as a companion of a patient. All healthy subjects signed an informed consent form. In addition to performing the MAT and completing the MFIQ, participants’ gender, age and number of OU were also noted.

### Masticatory performance

A comprehensive description of the MAT has been published previously [7, 14, 15]. The MAT enables the quantification of masticatory performance by assessing the patient’s ability to mix two wax layers of different colours (red and blue). The outcome variable of the MAT is the mixing ability index (MAI), where a MAI of 5 indicates mostly sufficient masticatory performance and a MAI of 30 indicates mostly insufficient masticatory performance. The test–retest reliability of the MAT is excellent (ICC = 0.906, 95% CI [0.801–0.957]) in condylar trauma patients [16]. The tablet consists of two 3-mm-thick layers of coloured Plasticine modelling wax (non-toxic DIN EN-71, art. nos. crimson 52,801 and blue 52,809; Stockmar, Kalten Kirchen, Germany) with a diameter of 20 mm. It is used at room temperature (20 °C) and forms a compact bolus during chewing. Subjects were instructed to chew on the tablet 15 times as if it were chewing gum. The chewed tablet was subsequently flattened to a thickness of 2.0 mm and photographed on both sides using a high-quality scanner (V750; Epson, Long Beach, CA, USA). The digitalised images were analysed and processed using a commercially available program for image analysis (Adobe Photoshop, CS3 extended; Adobe, San Jose, CA, USA). The MAI was obtained by measuring the intensity distribution of the red and blue colours on the combined image on both sides of the flattened wax.

### Masticatory ability

The MFIQ was designed to assess masticatory ability. The MFIQ has proven to be reliable in patients with painfully restricted TMJs (Spearman correlation of 0.69 to 0.96) [17]. The questionnaire consists of 17 items, comprising questions on speech, laughing, yawning and eating. Each item was answered using a 5-point Likert scale: 0, no difficulty; 1, a little difficulty; 2, quite a bit of difficulty; 3, a lot of difficulty; 4, very difficult or impossible without help. The total score ranges from 0 to 68, where 0 indicates no impairment of mandibular function and 68 indicates severely impaired mandibular function. The total outcome of the MFIQ was analysed as a continuous variable [17].

### Pain

To quantify pain, the validated VAS was used [18, 19]. Patients indicated their pain experience at rest at the time of examination by choosing a position on the 100-mm horizontal line, where 0 mm indicates no pain and 100 mm is the worst pain.

### Oral active range of motion

Maximum mouth opening (MMO) was measured twice intraorally as the distance between both maxillary and mandibular central incisors in the closed and maximal open positions. The greatest measured distance of overbite was added to the highest value of the two maximum mouth opening positions. Laterotrusion was measured as the distance between the midline of the central incisors of the maxilla and mandible. The value of the starting position in occlusion was added to or subtracted from the highest value of two measurements in maximum laterotrusion to each side. Protrusion was measured as the difference in distance between the labial side of the central incisors of the maxilla and mandible in occlusion and the highest value of two measurements in maximum forward protrusion.

### Statistical analysis

Categorical data is presented as frequency and percentages, whereas continuous data is expressed as the mean ± standard deviation (SD) and ordinal data as median ± interquartile range (IQR). For continuous data, normality was assessed using the Shapiro–Wilk test, as this is considered the most powerful test for data with non-normal distributions [20]. A linear regression analysis with MAI as well as MFIQ as the dependent outcome was constructed to assess the effect of characteristics in patients. We considered several potential associated factors: including gender, age, follow-up time, CDC, MMO, laterotrusion to ipsilateral side, laterotrusion to contralateral side, protrusion, TMJ symptoms, VAS_pain_ and number of OU. Thereafter, a linear regression analysis with MAI as well as MFIQ as the dependent outcome was constructed to assess the effect of characteristics in both patients and healthy subjects. We considered several potential associated factors: including gender, age, number of OU, and patient/healthy group (meaning if the subject was a patient or healthy subject). We performed unadjusted (i.e. for each variable separately) and adjusted linear regression analyses. Results were reported as regression coefficients with 95% CI and *P-*value. Data were analysed using SPSS version 25 (IBM Corporation, Armonk, NY, USA). *P*-values of 0.05 or less were considered statistically significant.

## Results

### Patients and healthy subjects

Ninety-five patients were identified based on the inclusion and exclusion criteria specified in the study protocol. Of these patients, only 82 were approached, as address information was unavailable for 13 cases. Forty-five responded, of which 23 expressed no interest in participating in this study. Upon further inquiry, one patient did not meet the inclusion criteria. In total, 21 patients with unilateral condylar neck (*N* = 9) or base (*N* = 12) fractures provided informed consent and were included in the study. The mean follow-up time was 11.67 (SD 4.89; range 5.11–22.30) years. Patient demographics and study outcomes are presented in Table 1. Several patients presented with secondary mandibular fractures. Of these patients, 12 received ORIF for fractures of the corpus and three for fractures of the mandibular angle. The mean duration of treatment was 7 weeks until discharge of the out-patient clinic. Maximum two weeks of the treatment were with wires or tight elastics, followed by guiding elastics until the patient reached maximal occlusion. In total, five patients presented complications according to the Clavien-Dindo classification: three patients required physiotherapy because of a limited range of motion after their initial treatment (grade I), and two patients reported altered occlusion that did not need any therapy (grade I). MAI and MFIQ outcomes are depicted in Table 2. Thirty healthy subjects were included in the study for comparison with the patient group. The subject characteristics, MAI and MFIQ outcome are also presented in Table 2.

**Table.1:** Demographics and study outcomes of included patients (*n* = 21)

*Demographics*		
Gender, *n (%)*		
Male	16	76.2
Female	5	23.8
Age, *mean (SD)*	47.43	15.97
Age at trauma, *mean (SD)*	35.76	13.55
Follow-up time, *mean (SD)*	11.67	4.89
Cause of trauma, *n (%)*		
Traffic	5	23.8
Bike	5	23.8
Violence	3	14.3
Fall	6	28.6
Sports	0	0
Other	2	9.5
Diagnose (AOCMF), *n (%)*		
Condylar neck fracture	9	42.9
Condylar base fracture	12	57.1
Other mandibular fractures, *n (%)*		
No	6	28.6
Yes	15	71.4
Type of MMF, *n (%)*		
Elastics	8	38.1
Wires followed by guiding elastics	13	61.9
Total duration of treatment in weeks, *mean (SD)*	7.0	2.5
Clavien-Dindo classification for complications		
Grade I	5	23.8
Grade II	0	0
Grade III a + b	0	0
Grade IV a + b	0	0
Grade V	0	0
No complications	16	76.2
***Study outcomes***		
MMO, *mean (SD)*	47.69	7.80
Laterotrusion, *mean (SD)*		
To ipsilateral side	10.33	4.08
To contralateral side	11.17	4.02
Protrusion, *mean (SD)*	8.33	3.24
TMJ symptoms, *n (%)*		
No	12	57.1
Yes	9	42.9
Occlusion, *n (%)*		
Stable	21	100
Patient-reported or objective malocclusion	0	0
Dentition status, *n (%)*		
Natural dentition	17	81
Partial denture	3	14
Maxillary denture, mandibular natural dentition	1	1
VAS for pain, *mean (SD)*	2.05	4.07

*AOCMF*, classification system for condylar process fractures; *MMF*, maxillomandibular fixation; *MMO*, maximum mouth opening; *SD*, standard deviation; *TMJ*, temporomandibular joint; *VAS*, Visual Analogue Scale

**Table.2:** Subject characteristics, MAI and MFIQ outcomes

	*Patient group n*** = *21***		*Healthy subjects n*** = *30***		
	*Mean (SD)*	*Median (IQR)*	*Mean (SD)*	*Median (IQR)*	
Gender*, n (%)*					
Male	16 (76.2)		19 (63.3)		
Female	5 (23.8)		11 (36.7)		
Age	47.43 (15.97)		34.20 (14.26)		
OU	9.52 (4.46)		11.9 (1.00)		
MAI	18.80 (2.27)		17.42 (1.76)		
MFIQ outcome	4.29 (6.22)		0.47 (1.14)		
1.Social activities	0.00 (0.00)	0 (0)	0.00 (0.00)	0 (0)	
2.Speaking	0.05 (0.22)	0 (0)	0.03 (0.18)	0 (0)	
3.Biting	0.67 (0.86)	0 (1)	0.03 (0.18)	0 (0)	
4.Eating hard food	0.38 (0.81)	0 (1)	0.07 (0.25)	0 (0)	
5.Eating soft food	0.19 (0.87)	0 (0)	0.00 (0.00)	0 (0)	
6.Daily activities	0.10 (0.30)	0 (1)	0.00 (0.00)	0 (0)	
7.Drinking	0.05 (0.22)	0 (0)	0.00 (0.00)	0 (0)	
8.Laughing	0.14 (0.48)	0 (0)	0.03 (0.18)	0 (0)	
9.Chewing resistant food	0.38 (0.67)	0 (1)	0.07 (0.25)	0 (0)	
10.Yawning	0.19 (0.40)	0 (0)	0.07 (0.25)	0 (0)	
11.Kissing	0.05 (0.22)	0 (0)	0.00 (0.00)	0 (0)	
12.Eating hard cookies	0.24 (0.63)	0 (0)	0.00 (0.00)	0 (0)	
13.Eating meat	0.24 (0.70)	0 (0)	0.03 (0.18)	0 (0)	
14.Eating raw carrot	0.38 (0.67)	0 (1)	0.03 (0.18)	0 (0)	
15.Eating French bread	0.19 (0.40)	0 (0)	0.07 (0.25)	0 (0)	
16.Eating peanuts	0.38 (0.67)	0 (1)	0.00 (0.00)	0 (0)	
17.Eating whole apple	0.67 (0.97)	0 (1)	0.03 (0.18)	0 (0)	

*MAI*, Mixing Ability Index; *MFIQ*, Mandibular Function Impairment Questionnaire; *OU*, occlusal units; *SD*, standard deviation; ^‡^ Mann–Whitney *U* test; ^†^ independent sample *T*-test; ^x^ chi square test; * *P* < 0.05; ** *P* < 0.01; *** *P* < 0.001

### Mastication and associated factors in patients

For the patient group, linear regression analyses are shown in Tables 3 and 4. In unadjusted linear regression analysis, the MAI was associated with the number of OU (*P* = 0.020). In the adjusted model, the number of OU remained significant (R^2^ = 0.253, Table 3). In unadjusted linear regression analysis, the MFIQ score was associated with gender (*P* = 0.039), VAS_pain_ (*P* = 0.001) and with OU (*P* = 0.030). In the adjusted model, the VAS_pain_ remained significant (R^2^ = 0.454, Table 4).

**Table.3:** Adjusted and unadjusted linear regression models for MAI in patients

Variable	Unadjusted model Regression coefficients (95% CI)	*P*-value	Adjusted model Regression coefficients (95% CI)	*P*-value
Gender	− 0.125 (− 2.621–2.371)	0.918		
Age (years)	− 0.021 (− 0.047–0.088)	0.529		
Follow-up time	0.055 (− 0.167–0.276)	0.661		
CDC	− 1.884 (− 4.211–0.443)	0.107		
MMO	− 0.120 (− 0.247–0.007)	0.063		
Ipsilateral laterotrusion	− 0.136 (− 0.396–0.123)	0.284		
Contralateral laterotrusion	− 0.158 (− 0.418–0.102)	0.217		
Protrusion	− 0.181 (− 0.506–0.144)	0.257		
TMJ symptoms	0.378 (− 1.763–2.512)	0.716		
VAS_pain_	0.144 (− 0.125–0.412)	0.275		
OU	− 0.256 (− 0.467– − 0.045)	0.020*	0.256 (− 0.467– − 0.045)	0.020*
MFIQ	0.082 (− 0.088–0.253)	0.326		
R^2^			0.253	

*CDC*, Clavien-Dindo Classification; *CI*, confidence interval; *MAI*, Mixing Ability Index; *MFIQ*, Mandibular Function Impairment Questionnaire; *MMO*, maximum mouth opening; *OU*, occlusal units; *TMJ*, temporomandibular joint; *VAS*, Visual Analogue Scale; * *P* < 0.05

**Table.4:** Adjusted and unadjusted linear regression models for MFIQ in patients

Variable	Unadjusted model Regression coefficients (95% CI)	*P*-value	Adjusted model Regression coefficients (95% CI)	*P*-value
Gender	6.450 (0.345–12.555)	0.039*		
Age (years)	0.075 (− 0.108–0.259)	0.401		
Follow-up time	0.497 (− 0.065–1.059)	0.080		
CDC	2.250 (− 4.510–9.010)	0.494		
MMO	0.028 (− 0.355–0.411)	0.881		
Ipsilateral laterotrusion	− 0.417 (− 1.122–0.288)	0.231		
Contralateral laterotrusion	− 0.343 (− 1.067–0.381)	0.334		
Protrusion	0.723 (− 0.130–1.577)	0.092		
TMJ symptoms	− 3.417 (− 9.075–2.242)	0.222		
VAS_pain_	1.056 (0.483–1.630)	0.001**	1.056 (0.483–1.630)	0.001**
OU	− 0.660 (− 1.251– − 0.069)	0.030*		
MAI	0.618 (− 0.665–1.901)	0.326		
R^2^			0.454	

*CDC*, Clavien-Dindo Classification; *CI*, confidence interval; *MAI*, Mixing Ability Index; *MFIQ*, Mandibular Function Impairment Questionnaire; *MMO*, maximum mouth opening; *OU*, occlusal units; *TMJ*, temporomandibular joint; *VAS*, Visual Analogue Scale; * *P* < 0.05; ** *P* < 0.01; *** *P* < 0.001

### Mastication and associated factors in both patients and healthy subjects

In unadjusted linear regression analysis for patients and healthy subjects, the variables age (*P* = 0.013), number of OU (*P* = 0.001), MFIQ (*P* = 0.028) and patient/healthy group (*P* = 0.019; meaning if subject was a patient or healthy subject) were associated with MAI (Table 5). The adjusted model showed that number of OU was associated with MAI (R^2^ = 0.201). In unadjusted linear regression analysis for MFIQ, the variables age (*P* = 0.023), number of OU (*P* = 0.000), MAI (*P* = 0.028) and patient/healthy group (*P* = 0.002) were of significant influence (Table 6). The adjusted model showed that number of OU and patient/healthy group remained explanatory variables for MFIQ (R^2^ = 0.343).

**Table.5:** Adjusted and unadjusted linear regression models for MAI in patients and healthy subjects

Variable	Unadjusted model Regression coefficients (95% CI)	*P*-value	Adjusted model Regression coefficients (95% CI)	*P*-value
Gender	0.718 (− 0.537–1.974)	0.256		
Age (years)	0.044 (0.010–0.079)	0.013*		
OU	− 0.296 (− 0.465– − 0.126)	0.001**	− 0.296 (− 0.465– − 0.126)	0.001**
MFIQ	0.144 (0.016–0.271)	0.028*		
Patient/healthy group	− 1.372 (− 2.505– − 0.239)	0.019*		
R^2^			0.201	

*CI*, confidence interval; *MAI*, Mixing Ability Index; *MFIQ*, Mandibular Function Impairment Questionnaire; *OU*, occlusal units; * *P* < 0.05; ** *P* < 0.01

**Table.6:** Adjusted and unadjusted linear regression models for MFIQ in patients and healthy subjects

Variable	Unadjusted model Regression coefficients (95% CI)	*P*-value	Adjusted model Regression coefficients (95% CI)	*P*-value
Gender	1.582 (− 1.108–4.273)	0.243		
Age (years)	0.087 (0.013–0.162)	0.023*		
OU	− 0.752 (− 1.096– − 0.408)	0.000***	− 0.612 (− 0.971– − 0.253)	0.001**
MAI	0.661 (0.075–1.247)	0.028*		
Patient/healthy group	− 3.819 (− 6.146–1.492)	0.002**	− 2.364 (− 4.639– − 0.090)	0.042*
R^2^			0.343	

*CI*, confidence interval; *MAI*, Mixing Ability Index; *MFIQ*, Mandibular Function Impairment Questionnaire; *OU*, occlusal units; * *P* < 0.05; ** *P* < 0.01; *** *P* < 0.001

## Discussion

This study assessed long-term explanatory demographic and clinical variables for masticatory performance and masticatory ability in patients who received closed treatment for unilateral condylar neck or base fractures. The patient group was examined 11.7 years on average after initial treatment. We also compared patients’ masticatory performance and ability with healthy subjects.

### Mastication and associated factors in patients

In patients with a history of unilateral condylar neck or base fractures, we found that the number of OU explained 25% of the masticatory performance, as R^2^ was 0.253. Further, patient-reported pain and dental status significantly influenced masticatory ability, as measured by the MFIQ, where patient-reported pain explained 45% of the masticatory ability as R^2^ was 0.454. These findings are consistent with a prospective clinical study of 114 patients with unilateral (73%) and bilateral (27%) condylar fractures who received MMF treatment [4]. Similar to our findings, the mean MFIQ outcome and the mean VAS_pain_ were 3.4 (SD 7.3) and 2.3 (SD 9.3), respectively, with a follow-up period of one year [4]. This indicates that there is negligible additional gain in patients’ perception of functional recovery after one year, which suggests that patients’ perception of rehabilitation reaches a plateau [4].

However, a prospective clinical study from 2006, with a follow-up period of 6 months, found poorer scores on the MFIQ (mean 10.5, SD 12.1) [21]. Although this is a worse outcome than our long-term result, the standard deviation was large and the follow-up period was short. Comparison with our results suggests that a 6-month follow-up period is too short, and improvement of masticatory ability after this period could be expected.

When comparing the mastication results from our patient group to those in the literature, similar results were reported in a cross-sectional study without a specified follow-up period in 48 patients with unilateral condylar fracture who received closed treatment [10]. In this study, both the MAT and MFIQ were performed (mean 18.4, SD 2.3; mean 4.96, SD 1.3, respectively), and a significant correlation was found (*r* = 0.250, *P* = 0.033) [10]. In this study, only the distinction between dentulous and edentulous patients was made; therefore, comparison to our results is difficult. In our patient group, the MAI and MFIQ did not remain a significant explanatory factor towards each other in adjusted analysis. This means that objective functionality does not necessarily correspond to patient-reported outcomes [21]. Therefore, objective and patient-reported measurements of functioning are complementary, and both results should be considered when deciding on the treatment that best meets the needs of the patient [22, 23].

The association between the number of OU and the MAI indicates the necessity of natural dentition with a sufficient amount of OU (> 4) for an optimal masticatory performance. Therefore, preservation and restoration of dentition and occlusal units are of great value in terms of masticatory performance and rehabilitation [24].

### Mastication and associated factors in both patients and healthy subjects

In this study, we found that the patient group had similar MAI outcomes compared to the healthy subjects when correcting for its explanatory variable OU as patient/healthy group did not remained an explanatory variable in the adjusted analysis. We found that OU was the influencing factor towards MAI for 20.1%, as R^2^ was 0.201. This is consistent with the literature where, as is known, dental state is one of the key determinants of masticatory performance [9]. In a recent study published in 2021, increasing number of OU significantly shortened the chewing time and therefore would increase the mixing ability [25]. This is consistent with the findings of a systematic review published in 2015, where the effects of removable dentures compensated for reduced masticatory performance in the order of 50% [24]. This could mean that a chewing cycle for the MAT of 15 strokes was insufficient for the denture wearers in our patient group to achieve the same outcome as people with natural dentition [7]. Our findings of masticatory performance when corrected for dentition status are therefore consistent with similar studies, which reported that mastication is equivalent to healthy subjects after 1 year of follow-up [26, 27]. Further, we found that the patient group had inferior masticatory ability, measured by the MFIQ, compared to healthy subjects as patient/healthy group remained an explanatory variable in adjusted analysis.

To the best of our knowledge, this is the first long-term study to use reliable methods to measure both masticatory performance and ability in the same patient group after MMF for unilateral condylar neck or base fractures. Throughout the study, there was strict compliance to the protocol. Demographic information of subjects who did not respond to the invitation to participate could not be compared to those evaluated at follow-up. Furthermore, despite approaching a large amount of subjects, only 51 participants were included in the study. This may influence the interpretation of our results.

Insufficient anatomical reduction of fractures after closed treatment could provide a functionally acceptable result for patients [28, 29]. Objectively measured oral functioning and patient-reported oral functioning can be complementary for treatment selection, even if they are not identical. Therefore, these measures should be combined in future research [22]. When anatomical measurements of the mandibular condyle are considered, it is possible to determine whether higher MAI or MFIQ outcomes are related to worse anatomical positioning after recovery. Prospective comparative studies are necessary to determine whether the treatment should remain focussed on objective outcomes or shift towards patient-reported outcomes to achieve the best results.

## Conclusions

In patients with a history of unilateral mandibular condylar neck or base fractures who received closed treatment, the number of OU is an explanatory factor for long-term masticatory performance, and patient-reported pain was an explanatory factor for masticatory ability. Long-term masticatory performance was similar in the patient group and healthy subjects; however, masticatory ability was inferior in the patient group. OU was of significant influence for both masticatory performance and masticatory ability.
